# Comparison between simple triage and rapid treatment and Taiwan Triage and Acuity Scale for the emergency department triage of victims following an earthquake-related mass casualty incident: a retrospective cohort study

**DOI:** 10.1186/s13017-020-00296-2

**Published:** 2020-03-11

**Authors:** Yun-Kuan Lin, Kuang-Yu Niu, Chen-June Seak, Yi-Ming Weng, Jen-Hung Wang, Pei-Fang Lai

**Affiliations:** 1 Department of Emergency Medicine, Hualien Tzu Chi Hospital, Buddhist Tzu Chi Medical Foundation, Hualien, Taiwan; 2 Department of Emergency Medicine, Lin-Kou Medical Center, Chang Gung Memorial Hospital and College of Medicine, Chang Gung University, Taoyuan, Taiwan; 3 Department of Emergency Medicine, Prehospital Care Division, Taoyuan General Hospital, Taoyuan, Taiwan; 4 Faculty of Medicine, National Yang-Ming University, Taipei, Taiwan; 5 Department of Medical Research, Hualien Tzu Chi Hospital, Buddhist Tzu Chi Medical Foundation, Hualien, Taiwan; 6 Department of Medicine, Tzu Chi University, Hualien, Taiwan

**Keywords:** Triage, Mass casualty incident, Earthquake, Emergency department triage

## Abstract

**Background:**

Triage plays a crucial role in the emergency department (ED) management of mass casualty incidents (MCIs) when resources are limited. This study aimed to compare the performance of simple triage and rapid treatment (START) with that of the Taiwan Triage and Acuity Scale (TTAS) for the ED triage of victims following an earthquake-related MCI.

**Methods:**

We retrospectively reviewed the records of victims presenting at our ED with earthquake-related injuries within 24 h of a large-scale earthquake. TTAS was initially used at our ED for this event, and START was performed by retrospectively reviewing the patient records in a blinded manner. Area under the receiver operating characteristic curve (AUC), sensitivity, and specificity of START and TTAS were determined for predicting ED discharge.

**Results:**

We enrolled 105 patients (predominantly women, 60.0%; median age, 45.0 years) in this study; most of them presented with traumatic injuries and were initially triaged as TTAS level III (78.1%), followed by TTAS level II (11.4%). Although the majority of the victims (81.0%) were discharged, four deaths occurred. A moderate agreement in differentiating emergency from nonemergency patients was observed between START and TTAS. Furthermore, both the triage systems showed similar predictions for ED disposition (START AUC/sensitivity/specificity: 0.709/82.35%/55.00%; TTAS AUC/sensitivity/specificity: 0.709/90.59%/45.00%).

**Conclusions:**

The present study demonstrated that START and TTAS have similar triage accuracy and ability to predict ED disposition. Our findings demonstrate that START may be used as an alternative to TTAS for the ED triage of victims following earthquake-related MCIs.

## Background

Field triage [1, 2] and hospital triage [3–5] play crucial roles in emergency medical care [6, 7]. The accuracy and efficiency of triage contribute to timely medical treatment and better patient outcomes [8]. However, inappropriate triage protocols may lead to catastrophic consequences, such as misusing valuable resources on overtriaged patients and jeopardizing undertriaged ones [9]. During disastrous scenarios, the efficiency of triage is particularly important for managing casualties when resources are limited [10].

Life-threatening disasters may lead to mass casualty incidents (MCIs), thereby further paralyzing regional health care resources and facilities [11]. While different disasters lead to different types of casualties, some of them such as explosions [12], fires [13], and traffic accidents [14] yield victims with relatively more predictable injuries. Large-scale earthquake-related disasters are characterized by a high number of victims with wide-range disease severity and injuries. The time frame and geographical casualty distribution of MCIs can be highly clustered around the epicenter, thus causing a surge in evacuations and medical demands [11, 15]. Owing to safety considerations, field triage and patient stabilization may not always be plausible, thereby leading to considerable challenges in the emergency department (ED) management of large-scale earthquake-related MCIs. Therefore, an optimal triage system that considers time and precision may improve resource allocation, maintain ED operability, and provide effective treatment to victims.

EDs throughout Taiwan use the Taiwan Triage and Acuity Scale (TTAS) [16], which has been adapted from the Canadian Triage and Acuity Scale (CTAS) [17], as the standard triage protocol to classify patients into five categories according to their acuity. However, TTAS is not specifically designed for situations wherein mass casualties are transported to EDs within a short duration. Using TTAS may result in time- and labor-consuming evaluation during disastrous MCIs, such as large-scale earthquakes. In the USA, simple triage and rapid treatment (START), which had been developed during the 1980s in Orange County, California, has been used as the *de facto* national standard for onsite triage during MCIs [8, 18]. The potential advantages of START include its simplicity, shorter triage time, and lower requirements for provider training [19]. Given that a large proportion of large-scale earthquake-related victims are transported to EDs without field triage [15], adopting START as an alternative triage protocol for the ED management of MCIs is plausible. However, studies demonstrating the use of START in ED settings have been limited, and its triage efficacy has not been verified to date [12, 15]. Indeed, comparing the performances of different triage protocols under the same setting remains an important field of study [20, 21].

On February 6, 2018, at 23:50 local time, a Richter 6.0 earthquake struck Hualien, a county of Eastern Taiwan, which ultimately caused hundreds to be injured, 17 deaths, and four complex building collapses. Government estimates revealed that the earthquake caused over 250 million USD damage. Considering that TTAS had been initially used by our ED for this event, the present study performed START by retrospectively reviewing patient records in a blinded manner and compare the performances of START and TTAS for the emergency department triage of victims following an earthquake-related mass casualty incident. Accordingly, we found that START and TTAS had similar triage accuracy and ability to predict ED disposition.

## Methods

### Study design and setting

This study was approved by the Institutional Review Board of the Hualien Tzu Chi Hospital Research Ethics Committee, and the need for informed consent was waived. This study was conducted at a tertiary center of Eastern Taiwan, which comprises 970 hospital beds and 76 ED beds. Victims of the Hualien earthquake (occurred on February 6, 2018) who visited our ED were originally categorized according to different acuity levels using the standard TTAS protocol. Records of victims who presented at our ED within 24 h after the earthquake were retrospectively reviewed. All the patients who experienced earthquake-related injuries, including traumas, burns, inhalation injuries, and out-of-hospital cardiac arrest, were included, whereas young patients (aged < 8 years), those who arrived late (arriving > 24 h after the earthquake), and patients with incomplete vital sign records were excluded. The included victims were then re-evaluated using START by two ED clinicians blinded to the TTAS outcomes.

### Taiwan Triage and Acuity Scale assessment

TTAS [16], a computerized triage system adapted from CTAS [17], has been the standard emergency triage protocol used in Taiwan. The TTAS level for each patient was generated in real-time by one designated triage nurse who had received specific training on the application of the five-level TTAS protocol and the computer-assisted system. Using the computerized decision support system, patients were classified in descending order of acuity: level 1, resuscitation; level 2, emergency; level 3, urgent; level 4, less urgent; and level 5, nonurgent. TTAS categorizes patients into three domains: nontrauma, trauma, or environmental injuries. The nontrauma domain includes 13 categories and 125 chief complaints, the trauma domain includes 14 categories and 41 chief complaints, and the environmental injury domain includes 11 chief complaints. TTAS determines triage severity according to (a) chief complaints and (b) first-order modifiers such as vital signs (including respiration, hemodynamics, consciousness level, and body temperature), pain severity, and injury mechanism (for trauma patients). Second-order modifiers are used when first-order modifiers are unable to adequately assign an appropriate acuity level and are specific to a few complaints such as visual disturbance for eye trauma or neurologic deficit for head, neck, and back trauma.

### Simple triage and rapid treatment assessment

**Fig. 1:**
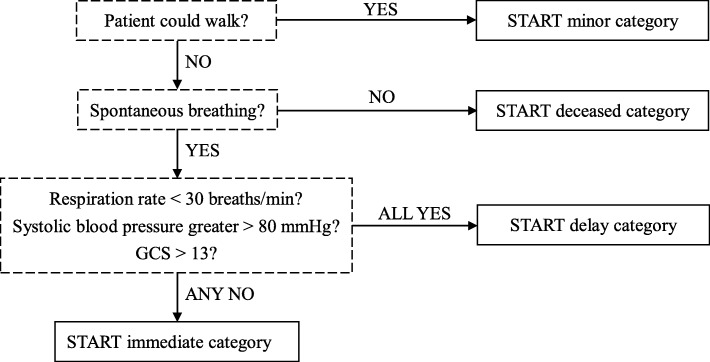
Simple triage and rapid treatment algorithm. *GCS* Glasgow Coma Scale, *START* simple triage and rapid treatment

START acuity was retrospectively determined using recorded ambulatory status, respiratory rate, pulse, and consciousness level. Accordingly, patients who could walk were assigned to the START minor category, those unable to breathe spontaneously were assigned to the deceased category, and those who could not walk, had a respiratory rate of < 30 breaths/min and systolic blood pressure of > 80 mmHg, and were able to follow commands (Glasgow Coma Scale score > 13) were assigned to the delay category. All the other patients were assigned to the immediate category (Fig. 1). Ambiguous cases were clarified and assigned after consensus among the reviewing investigators during data abstraction.

### Data collection and processing

Demographic data, including age, sex, TTAS level, ED disposition [discharge, observations, admission to the ward or intensive care unit (ICU), and ED mortality], ED interventions, ED length of stay (LOS), mortality, and ED medical expenses, were obtained. Specific ED interventions analyzed herein included transfusion of blood products and computed tomography (CT) arrangement. ED medical expenses were determined from the Medical Affairs Office of the hospital, excluding the costs of prehospital care, after ED discharge or admission. Patients were further classified into emergency and nonemergency groups based on either START or TTAS levels. Accordingly, patients in the START immediate and deceased categories were classified into the emergency group, indicating that standard resuscitation was performed on deceased patients, whereas those in the minor and delay categories were classified into the nonemergency group. Moreover, patients triaged as TTAS levels I and II were classified into the emergency group, whereas those triaged as levels III–V were as classified into the nonemergency group.

### Outcome measures

The primary outcome was ED discharge rate, whereas the secondary outcomes included ED interventions, ED LOS, 24-h mortality, and ED medical expenses.

### Statistical analysis

The Kolmogorov–Smirnov test was used to determine the distribution of continuous variables. Continuous variables were presented as medians [interquartile ranges (IQRs)] and compared using the Kruskal–Wallis test, whereas categorical variables were presented as percentages and compared using the chi-squared or Fisher’s exact test. Kappa statistic was used to describe the consistency between START and TTAS in defining emergency or nonemergency patients. Sensitivity, specificity, positive predictive value, negative predictive value, and area under the receiver operating characteristic (ROC) curve (AUC) of START and TTAS for predicting ED discharge were calculated. A *p* value of < 0.05 indicated statistical significance. Statistical analyses were performed using SPSS (IBM SPSS Statistics for Windows, Version 21.0, Armonk, NY: IBM Corp.) and MedCalc (MedCalc for Windows, Version 19.1, Ostend, Belgium).

## Results

**Fig. 2:**
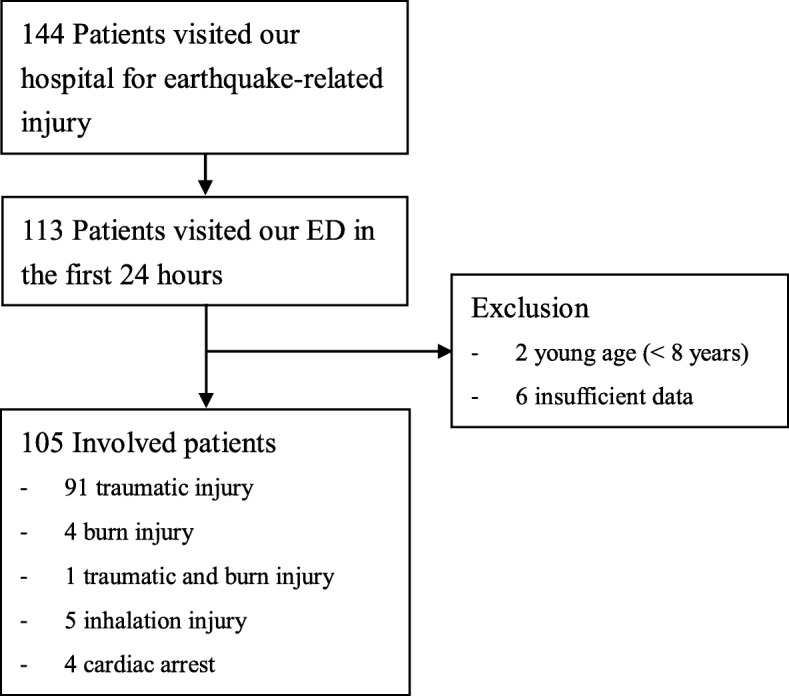
Flow diagram of the study. *ED* emergency department

A total of 144 patients visited the ED at our hospital after the earthquake. Among these, 113 visited our ED within the first 24 h of the earthquake. After excluding 2 patients aged < 8 years and 6 patients with incomplete vital sign records, a total of 105 patients were finally included for analysis (Fig. 2). Most of the patients developed traumatic injuries (*n* = 92), whereas the others developed burn injuries (*n* = 5, 1 of whom also developed trauma), inhalation injury (*n* = 5), and cardiac arrest (*n* = 4). Table 1 presents the demographic characteristics of the study population. Our patients were predominantly women (60.0%) with a median age of 45.0 years. Most of them were initially triaged as TTAS level III (78.1%), followed by TTAS level II (11.4%). Most of the victims were discharged (81.0%), whereas only 4 deaths were noted. The majority of the patients triaged using the START protocol were classified into the minor category, followed by the delay, deceased, and immediate categories. Patients under the START minor category were younger and had higher discharge rates and lower ED medical expenses than those in the other categories. Patients in the START delay category had a higher probability of undergoing CT at the ED than those in the START minor category (38.1% vs. 10.1%; *p* = 0.002). Only 1 and 2 patients in the delay (4.8%) and deceased (50%) categories, respectively, received blood transfusion at the ED. Deaths were only observed in the deceased category, with 3 and 1 patient dying during their ED and ICU stays, respectively. These four mortality cases were categorized as TTAS level I and were declared out-of-hospital cardiac arrest/dead on arrival. One patient who died had suddenly collapsed shortly after the earthquake without trauma, whereas the other three patients were rescued from a collapsed building with head and/or extremity trauma.

**Table 1:** Comparison of patient characteristics and outcomes according to START triage categories

	Overall	Minor category	Delay category	Immediate category	Deceased category	*p* value
	*n* = 105	*n* = 79 (75.2%)	*n* = 21 (20.0%)	*n* = 1 (1.0%)	*n* = 4 (3.8%)	
Sex, *n* (%)						0.84
Male	42 (40.0)	33 (41.8)	7 (33.3)	0 (0)	2 (50)	
Female	63 (60.0)	46 (58.2)	14 (66.7)	1 (100)	2 (50)	
Age, years, median (IQR)	45.0 (35.0)	38.0 (32.0)	59.0 (29.5)	80	49.5 (27.8)	0.008*
ED disposition, *n* (%)						< 0.001*
ED mortality, *n* (%)	3 (2.9)	0 (0)	0 (0)	0 (0)	3 (75)	
ICU, *n* (%)	2 (1.9)	0 (0)	1 (4.8)	0 (0)	1 (25)	
Ward, *n* (%)	8 (7.6)	5 (6.3)	3 (14.3)	0 (0)	0 (0)	
Observation, *n* (%)	7 (6.7)	4 (5.1)	2 (9.5)	1 (100)	0 (0)	
Discharge, *n* (%)	85 (81.0)	70 (88.6)	15 (71.4)	0 (0)	0 (0)	
CT, *n* (%)	18 (17.1)	8 (10.1)	8 (38.1)	1 (100)	1 (25)	0.002*
Blood transfusion, *n* (%)	3 (2.9)	0 (0)	1 (4.8)	0 (0)	2 (50)	0.001*
ED LOS in hours, median (IQR)	1.02 (1.02)	0.98 (0.89)	1.18 (2.41)	20.03	0.88 (0.41)	0.134
Mortality, *n* (%)	4 (3.8)	0 (0)	0 (0)	0 (0)	4 (100)	< 0.001*
ED medical expenses, median (IQR), USD	122.7 (164.1)	116.6 (83.4)	173.0 (484.5)	1150	552.7 (327.7)	0.003*
TTAS						< 0.001*
Level I	5 (4.8)	0 (0)	1 (4.8)	0 (0)	4 (100)	
Level II	12 (11.4)	9 (11.4)	2 (9.5)	1 (100)	0 (0)	
Level III	82 (78.1)	66 (83.5)	16 (76.2)	0 (0)	0 (0)	
Level IV	6 (5.7)	4 (5.1)	2 (9.5)	0 (0)	0 (0)	
Level V	0 (0)	0 (0)	0 (0)	0 (0)	0 (0)	

*START* simple triage and rapid treatment, *IQR* interquartile range, *ED* emergency department, *ICU* intensive care unit, *CT* computed tomography, *LOS* length of stay, *USD* United States dollar, *TTAS* Taiwan Triage and Acuity Scale

After analyzing the consistency between START and TTAS in differentiating emergency from nonemergency patients, our results revealed a moderate agreement between the two triage systems (Cohen’s Kappa coefficient = 0.41; Table 2). Only 12 patients triaged into the emergency group (level I or II) by TTAS were triaged into the nonemergency group (minor or delay category) by START.

**Table 2:** Association between START and TTAS

		START			
		Nonemergency		Emergency	
		Minor category	Delay category	Immediate category	Deceased category
TTAS					
Nonemergency	Level V	**0**	**0**	0	0
	Level IV	**4**	**2**	0	0
	Level III	**66**	**16**	0	0
Emergency	Level II	9	2	**1**	**0**
	Level I	0	1	**0**	**4**

*START* simple triage and rapid treatment, *TTAS* Taiwan Triage and Acuity Scale

**Fig. 3:**
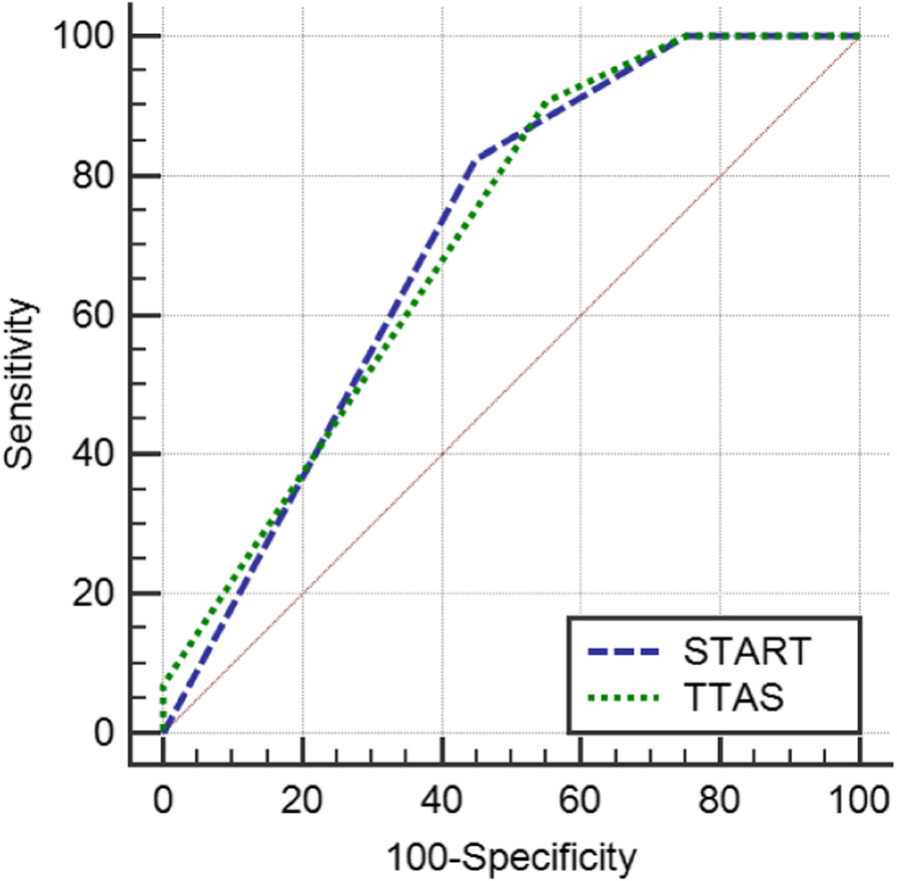
ROC curve determining the ability of START and TTAS to predict emergency department discharge. *START* simple triage and rapid treatment, *TTAS* Taiwan Triage and Acuity Scale, *ROC* receiver operating characteristic

ROC analysis was performed to determine the abilities of START and TTAS to predict ED discharge among our patients (Fig. 3). Accordingly, the AUC for START [0.709 (0.612–0.793)] was not significantly different from that for TTAS [0.709 (0.612–0.794); *p* = 0.996]. Table 3 summarizes the sensitivity and specificity of START and TTAS for predicting ED discharge. Accordingly, our results showed that START and TTAS had similar performances (START sensitivity/specificity: 82.35%/55.00%; TTAS sensitivity/specificity: 90.59%/45.00%). Similarly, START and TTAS had similar performances in predicting ED mortality (START AUC/sensitivity/specificity: 0.995/100%/99.02%; TTAS AUC/sensitivity/specificity: 0.990/100%/98.04%).

**Table 3:** START and TTAS protocols for predicting disposition (ED discharge vs. observation/admission/death)

	START		TTAS	
	Minor category	Delay/immediate/deceased category	Levels III–V	Levels I and II
ED discharge, *n*	70	15	77	8
Observation/admission/death, *n*	9	11	11	9
AUC, 95% CI	0.709 (0.612–0.793)		0.709 (0.612–0.794)	
Sensitivity, 95% CI	82.35 (72.57–89.77)		90.59 (82.29–95.85)	
Specificity, 95% CI	55.00 (31.53–76.94)		45.00 (23.06–68.47)	
PPV, 95% CI	88.61 (82.59–92.73)		87.50 (82.40–91.28)	
NPV, 95% CI	42.31 (28.56–57.36)		52.94 (33.17–71.83)	
Accuracy, 95% CI	77.14 (67.93–84.77)		81.90 (73.19–88.74)	

*START* simple triage and rapid treatment, *TTAS* Taiwan Triage and Acuity Scale, *ED* emergency department, *AUC* area under the receiver operating characteristic curve, *CI* confidence interval, *PPV* positive predictive value, *NPV* negative predictive value

## Discussion

The present study aimed to compare the performances of START and TTAS in managing the ED triage of victims following an earthquake-related MCI. Our results showed a moderate agreement between START and TTAS in differentiating emergency from nonemergency patients. Furthermore, both the triage systems had similar predictions regarding ED disposition.

Victims of earthquake-related disasters may present with a wide spectrum of injuries and severities [22–24]. In the present study, traumatic injuries were predominant, followed by burns and inhalation injuries. Earthquake-related MCIs may cause considerable challenges related to workforce and medical resources within the ED. During such situations, a rapid and accurate triage system is essential for the subsequent management and improvement of patient outcomes. Despite being conventionally used in EDs, TTAS had not been specifically designed for the triage of MCI victims and therefore requires evaluations involving time-consuming, labor-intensive, computer-assisted processes, which may cause delays in patient management following MCIs. Moreover, safety considerations during an earthquake may prevent field triage from being performed. Thus, the use of the START system appears feasible for the ED management of victims of earthquake-related MCIs. Indeed, the findings of our study demonstrated that both START and TTAS had similar triage accuracy and ability to predict ED disposition.

A limited number of studies have compared the performance of START with those of other triage systems in an ED setting. Ng et al. [12] conducted a retrospective study using data obtained from casualties following a mass burn incident. After comparing the performances of START, TTAS, and a mass burn casualty triage system at the ED, they found that STRAT had the highest sensitivity but the lowest specificity in predicting ICU admission [12]. Curran-Sills and Franc [19], who investigated the speed and accuracy of START and CATS (the original version of TTAS) for the ED triage of patients following a simulated MCI, found that triage nurses completed the START protocol (33 s/patient) faster than they completed the CTAS protocol (138 s/patient), with both the systems showing similar levels of accuracy [19]. Moreover, after comparing the Emergency Severity Index triage levels and START colors for urgent care and hospitalization in a triage tag exercise, Hong et al. [25] found that the Emergency Severity Index better identified patients with abnormal vital signs, those who needed emergency interventions, and those who needed hospitalization than START [25]. To the best of our knowledge, the present study is the first to compare the performances of START and TTAS in the triage of victims transported to the ED following a large-scale earthquake.

START had been developed for resource-limited field triage settings, prioritizing patients in the START immediate category who are more probable to survive rather than those in the deceased category. Conversely, TTAS had been designed as a triage system for patients arriving at EDs, prioritizing the most severe patients (TTAS level I patients). The present study demonstrated that victims in the deceased category had a 100% mortality rate even after standard in-hospital resuscitation efforts and higher medical expenses compared with those in the other categories. Nevertheless, we suggest that patients in the START deceased category be prioritized when the aforementioned triage system is alternatively used for evaluation in EDs.

Among the 12 patients with discrepant triage results (emergency by TTAS but nonemergency by START), 9 had head injuries, whereas 3 had upper extremity injuries. Among those with head injuries, an 87-year-old male patient diagnosed with a brain concussion had been admitted to the ICU for further observation over 3 days. This patient was triaged as TTAS level I and retrospectively classified in the START delay category, whereas the remaining 8 patients had been discharged from the ED. Although concerns have been raised regarding the possibility of overtriage with START [26, 27], the present study demonstrated no such tendency after comparing the results for START and TTAS.

Large-scale earthquake-related MCIs are rare, but they substantially increase the difficulty in the triage and management of victims at EDs. Therefore, the data presented in the present study may be of considerable value. However, some limitations of this study are noteworthy. First, given the retrospective nature of this study and the small number of patients included, selection bias may have affected our results. Second, the effects of certain confounders such as triage nurse experience and potential factors influencing physician judgment have not been determined. Third, this study was conducted at a university-affiliated teaching medical center following a large-scale earthquake, which might limit the generalizability of our findings. Therefore, further retrospective or prospective research is needed to validate the use of START at EDs when large sample sizes of patients following either large-scale earthquakes or other MCIs are encountered in the future. However, a prospective randomized study using the START and TTAS triage systems at EDs following MCIs is feasible only with both the validation of the use of START by more retrospective observations and the careful consideration of ethical issues. If randomized studies yield promising results, START may be considered as the main triage system for victims of large-scale earthquakes or other MCIs.

## Conclusions

The present study demonstrated that START and TTAS had similar triage accuracy and ability to predict ED disposition. Given that START allows shorter triage times compared with TTAS, our findings suggest that START is an alternative to TTAS for the ED triage of victims of earthquake-related MCIs. However, the findings of our study need to be elucidated in further investigations.

## Data Availability

The datasets used and analyzed during the current study are available from the corresponding author on reasonable request.

## Abbreviations

AUC: Area under the ROC curve
CI: Confidence interval
CT: Computed tomography
CTAS: Canadian Triage and Acuity Scale
ED: Emergency department
ICU: Intensive care unit
IQR: Interquartile range
LOS: Length of stay
MCI: Mass casualty incident
ROC: Receiver operating characteristic
START: Simple triage and rapid treatment
TTAS: Taiwan Triage and Acuity Scale

## References

[1] Sasser SM, Hunt RC, Faul M, Sugerman D, Pearson WS, Dulski T, Wald MM, Jurkovich GJ, Newgard CD, Lerner EB, Cooper A (2012). Guidelines for field triage of injured patients: recommendations of the national expert panel on field triage, 2011. MMWR Recomm Rep Morb Mortal Wkly Rep Recomm Rep.

[2] McCoy CE, Chakravarthy B, Lotfipour S (2013). Guidelines for field triage of injured patients: in conjunction with the Morbidity and Mortality Weekly Report published by the Center for Disease Control and Prevention. West J Emerg Med.

[3] Christ M, Grossmann F, Winter D, Bingisser R, Platz E (2010). Modern triage in the emergency department. Dtsch Arztebl Int.

[4] FitzGerald G, Jelinek GA, Scott D, Gerdtz MF (2010). Emergency department triage revisited. Emerg Med J.

[5] Farrohknia N, Castrén M, Ehrenberg A, Lind L, Oredsson S, Jonsson H, Asplund K, Göransson KE (2011). Emergency department triage scales and their components: a systematic review of the scientific evidence. Scand J Trauma Resusc Emerg Med.

[6] Iserson KV, Moskop JC (2007). Triage in medicine, part I: concept, history, and types. Ann Emerg Med.

[7] Moskop JC, Iserson KV (2007). Triage in medicine, part II: Underlying values and principles. Ann Emerg Med.

[8] Timbie JW, Ringel JS, Fox DS, Pillemer F, Waxman DA, Moore M, Hansen CK, Knebel AR, Ricciardi R, Kellermann AL (2013). Systematic review of strategies to manage and allocate scarce resources during mass casualty events. Ann Emerg Med.

[9] Zachariasse JM, van der Hagen V, Seiger N, Mackway-Jones K, van Veen M, Moll HA (2019). Performance of triage systems in emergency care: a systematic review and meta-analysis. BMJ Open.

[10] Bazyar J, Farrokhi M, Khankeh H (2019). Triage systems in mass casualty incidents and disasters: a review study with a worldwide approach. Open Access Maced J Med Sci.

[11] Merin O, Ash N, Levy G, Schwaber MJ, Kreiss Y (2010). The Israeli field hospital in Haiti—ethical dilemmas in early disaster response. N Engl J Med.

[12] Ng CJ, You SH, Wu IL, Weng YM, Chaou CH, Chien CY, Seak CJ (2018). Introduction of a mass burn casualty triage system in a hospital during a powder explosion disaster: a retrospective cohort study. World J Emerg Surg.

[13] Koning SW, Ellerbroek PM, Leenen LP (2015). Indoor fire in a nursing home: evaluation of the medical response to a mass casualty incident based on a standardized protocol. Eur J Trauma Emerg Surg.

[14] Postma IL, Weel H, Heetveld MJ, van der Zande I, Bijlsma TS, Bloemers FW, Goslings JC (2013). Mass casualty triage after an airplane crash near Amsterdam. Injury.

[15] Nie H, Tang SY, Lau WB, Zhang JC, Jiang YW, Lopez BL, Ma XL, Cao Y, Christopher TA (2011). Triage during the week of the Sichuan earthquake: a review of utilized patient triage, care, and disposition procedures. Injury.

[16] Ng CJ, Yen ZS, Tsai JC, Chen LC, Lin SJ, Sang YY, Chen JC (2011). TTAS national working group. Validation of the Taiwan triage and acuity scale: a new computerised five-level triage system. Emerg Med J.

[17] Bullard MJ, Musgrave E, Warren D, Unger B, Skeldon T, Grierson R, van der Linde E, Swain J (2017). Revisions to the Canadian Emergency Department Triage and Acuity Scale (CTAS) guidelines 2016. CJEM.

[18] Benson M, Koenig KL, Schultz CH (1996). Disaster triage: START, then SAVE—a new method of dynamic triage for victims of a catastrophic earthquake. Prehosp Disaster Med.

[19] Curran-Sills G, Franc JM (2017). A pilot study examining the speed and accuracy of triage for simulated disaster patients in an emergency department setting: comparison of a computerized version of Canadian Triage Acuity Scale (CTAS) and Simple Triage and Rapid Treatment (START) methods. CJEM.

[20] Bhalla MC, Frey J, Rider C, Nord M, Hegerhorst M (2015). Simple triage algorithm and rapid treatment and sort, assess, lifesaving, interventions, treatment, and transportation mass casualty triage methods for sensitivity, specificity, and predictive values. Am J Emerg Med.

[21] Tsai LH, Huang CH, Su YC, Weng YM, Chaou CH, Li WC, Kuo CW, Ng CJ (2017). Comparison of prehospital triage and five-level triage system at the emergency department. Emerg Med J.

[22] Del Papa J, Vittorini P, D’Aloisio F, Muselli M, Giuliani AR, Mascitelli A, Fabiani L. Retrospective analysis of injuries and hospitalizations of patients following the 2009 earthquake of L'Aquila City. Int J Environ Res Public Health. 2019;16. 10.3390/ijerph16101675 PMC6571652 31091681

[23] Pan ST, Cheng YY, Wu CL, Chang RH, Chiu C, Foo NP, Chen PT, Wang TY, Chen LH, Chen CJ, Ong R, Tsai CC, Hsu CC, Hsieh LW, Chi CH, Lin CH (2019). Association of injury pattern and entrapment location inside damaged buildings in the 2016 Taiwan earthquake. J Formos Med Assoc.

[24] Moitinho de Almeida M, JAF v L, Thapa SS, Kumar KC, Schlüter BS, Singh R, Banse X, Putineanu D, Mahara DP, Guha-Sapir D (2019). Clinical and demographic profile of admitted victims in a tertiary hospital after the 2015 earthquake in Nepal. PLoS One.

[25] Hong R, Sexton R, Sweet B, Carroll G, Tambussi C, Baumann BM (2015). Comparison of START triage categories to emergency department triage levels to determine need for urgent care and to predict hospitalization. Am J Disaster Med.

[26] Kahn CA, Schultz CH, Miller KT, Anderson CL (2009). Does START triage work? An outcomes assessment after a disaster. Ann Emerg Med.

[27] Hoff JJ, Carroll G, Hong R (2017). Presence of undertriage and overtriage in simple triage and rapid treatment. Am J Disaster Med.

